# Transition metal-ion mediated sulfur redox chemistry for aqueous batteries

**DOI:** 10.1093/nsr/nwad021

**Published:** 2023-02-01

**Authors:** Mingliang Yu, Xiulei Ji

**Affiliations:** Department of Chemistry, Oregon State University, USA; Department of Chemistry, Oregon State University, USA

Aqueous batteries represent promising solutions for grid-scale energy storage with the benefits of low-cost and high safety [1,2]. Tremendous efforts have been devoted to identifying high-capacity cathode materials to couple the zinc metal anode [3]. Sulfur exhibits a high theoretical specific capacity by transferring two electrons per atomic mass of 32.065 in forming a sulfide ion. In nonaqueous electrolytes, the operation potentials of the sulfur electrode are often maligned being below par at –0.9 V *vs* standard hydrogen electrode (SHE), where alkali metal ions serve as the charge carriers. However, when coupling with transition-metal ion charge carriers, e.g. Mn^2+^, Fe^2+^, Ni^2+^, Pb^2+^, and Cu^2+^, the reduction of sulfur to the corresponding metal sulfides exhibit much higher potentials [4–6]. Such operation potentials render sulfur electrodes compatible with common aqueous electrolytes and confer the S||Zn full-cell a competitive voltage.

**Figure 1.**
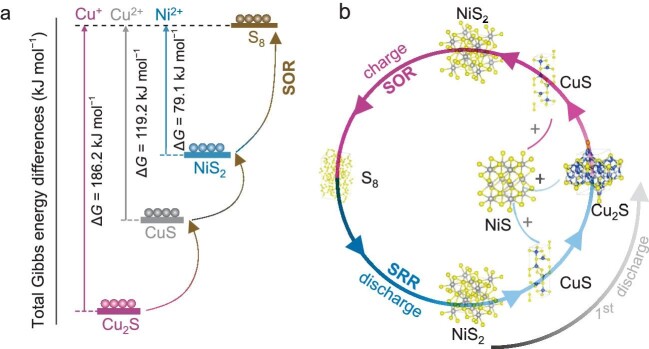
(a) The Gibbs energy changes of different conversation intermedium in the sulfur oxidation reaction process (SOR). (b) A schematic illustration of phase conversion during the cycling process.

However, challenges exist in the reversible oxidation of S^2−^ to S, where the efficiency had veered from unity due to the intrinsic thermodynamic instability and sluggish conversion kinetics. The sulfur oxidation reaction (SOR) efficiency is essential to the actual reversible capacity of the sulfur-based aqueous batteries. Writing in *National Science Review*, Zhao, Chao, and co-workers reported a ‘mesocrystal NiS_2_ mediating’ strategy, which showed a high SOR efficiency of ∼96.0% [7]. Characterization and calculations suggest the higher sulfur oxidation efficiency pertains to the lower energy barrier for NiS_2_ oxidation to S (Fig. 1a), favorable ionic diffusion, and high charge transfer. Benefiting from the activated SOR process, a 6e^−^ involved sulfur conversion (2S + Ni^2+^ + 2e^−^ ↔ NiS_2_, NiS_2_ + Cu^2+^ + 2e^−^ ↔ NiS + CuS, CuS + Cu^2+^ + 2e^−^ ↔ Cu_2_S) was demonstrated (Fig. 1b), and the mesocrystal NiS_2_ exhibited a high reversible capacity (1258 mAh g^−1^), superior rate capability (932 mAh g^−1^ at 12 A g^−1^), and long-term cyclability (2000 cycles at 20 A g^−1^). The new electrochemical reaction employed both Ni^2+^ and Cu^2+^ as the charge carriers for the charge neutrality compensation of the sulfur electrode. The presence of Ni^2+^ intermedium is essential for achieving the high Coulombic efficiency of the reversible reaction.

This study suggests that via the transition metal ion intermediate, the reaction route of an electrochemical reaction can be tailor-designed to suppress undesirable parasitic reactions and achieve higher SOR efficiency. The Li-S/Na-S community may use the concept of this work to avoid the dissolution and shuttling of intermediate polysulfides.
